# Low-vacuum scanning electron microscopy for informative three-dimensional imaging of cell/tissue architectures and biomedical target localization

**DOI:** 10.1093/jmicro/dfag012

**Published:** 2026-02-19

**Authors:** Akira Sawaguchi

**Affiliations:** Division of Ultrastructural Cell Biology, Department of Anatomy, Faculty of Medicine, University of Miyazaki, Miyazaki 889-1692, Japan

**Keywords:** low-vacuum, scanning electron microscopy, permanganate, gold labelling, uranium-free, paraffin/cryostat section

## Abstract

This review focusses on the recent advances of practical application of low-vacuum scanning electron microscopy to informative three-dimensional imaging of cell/tissue architectures and biomedical target localization on microscope slides for biomedical sciences and clinical diagnoses. Scanning electron microscopy under low-vacuum conditions allows high-resolution imaging of complex cell/tissue architectures in nonconductive specimens because the negative charge that accumulates on the nonconductive materials can be neutralized by the positive ions in the residual gas molecules. However, the conventional methods for metal staining of biological specimens require harmful uranium compounds, which hampers the applications of electron microscopy. The development of uranium-free KMnO_4_/Pb metal staining allows multiscale imaging of extensive cell/tissue architectures to intensive subcellular ultrastructure. The obtained image contrast was equivalent to that of Ur/Pb staining and sufficient for ultrastructural observation. Observation of the 20-µm-thick section facilitates distinctive perception of the face-side images of the epithelium, which are seldom seen within 5-µm-thin sections. Visualization of the exact location of targeting molecules by *in situ* strategy provides unique insight into nanogold development via nanogold nucleation and secondary growth under hot-humid air conditions. These user-friendly techniques are highly anticipated to fill the gap between light and electron microscopy to correlate cell/tissue structure and function. Importantly, paraffin and cryostat blocks of cell or tissue samples are semipermanent, making them valuable for retrospective studies through the re-evaluation of archived specimens.

## Teaser

This review focus on the recent advances of practical application of low-vacuum SEM to informative three-dimensional imaging of cell/tissue architectures and biomedical target localization for biomedical sciences and clinical diagnoses. These user-friendly techniques are anticipated to fill the gap between light and electron microscopy to correlate cell/tissue structure and function.

## Introduction

A key objective of biomedical imaging is to reveal how the structure of cells and tissues relates to their function. Electron microscopy is widely recognized as one of the most powerful techniques for investigating ultrastructures [1,2]. Over the past decade, numerous cell biologists have employed immunocytochemistry in conjunction with super-resolution fluorescence microscopy. Despite advancements in these imaging techniques, super-resolution microscopy remains fundamentally dependent on fluorescence labelling. In this technical context, electron microscopy is an essential method that continues to advance with new devices and techniques. It enables the correlation of cell and tissue structure with biological function [3,4]. Here, this review explores the recent advances in the low-vacuum scanning electron microscopy (Low-vac SEM) applied to biomedical sciences and clinical diagnoses, focusing on the advantages of the Low-vac SEM for nonconductive paraffin/cryostat section.

## Mechanical advantages of the Low-vac SEM for nonconductive paraffin/cryostat section

**Fig. 1.**
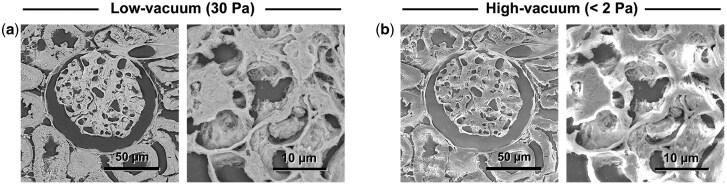
Advantages of the low-vacuum condition for nonconductive bio-medical samples. Low-vac SEM images of the renal corpuscle without charge-up obstruction in low-vacuum mode at 30 Pa (a), compared with high-vacuum condition lower than 2 Pa (b).

**Fig. 2.**
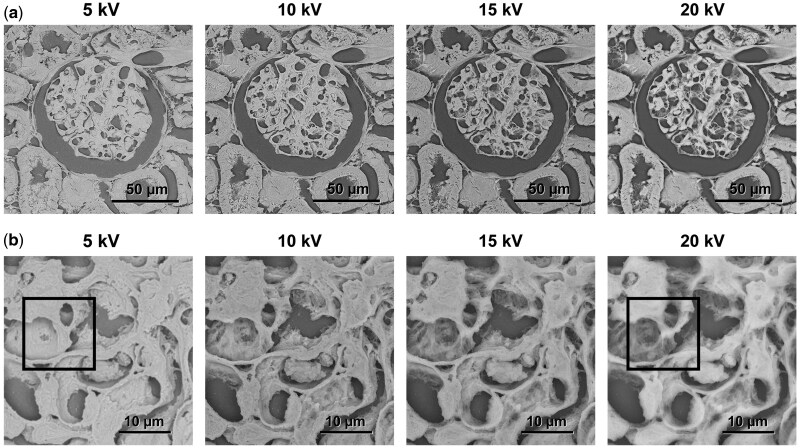
Tuning the acceleration voltage for Low-vac SEM imaging by KMnO_4_/Pb metal staining. Representative Low-vac SEM images at acceleration voltages of 5, 10, 15 and 20 kV. (a) Low-power view of the renal corpuscle. (b) High-power views of the glomerulus. Note the trade-off between the low contrast revealing surface undulations at 5 kV and the high contrast with transparency missing surface undulations at 20 kV within the boxed area.

Scanning electron microscopy (SEM) offers three-dimensional characterization of specimen surfaces by detecting electrons reflected from the surface (backscattered electrons, BSEs) and electrons forced out of the surface (secondary electrons, SEs). Low-vac SEM enables BSE and SE imaging of non-conductive biological samples by neutralizing surface charge with positive ions from residual gas molecules (Fig. 1) [5–7]. All Low-vac SEM images presented in this review were obtained with a BSE detector installed in the TM4000Plus II (Hitachi High-Tech, Tokyo, Japan). For fine structural observation, the physical property of the electron beam required us to tune the acceleration voltage according to the magnification of the target structure [8]. Comparative electron micrographs (Fig. 2) clarify the trade-off between the low contrast revealing surface undulations at 5 kV and the high contrast missing surface undulations at 20 kV. The use of a lower accelerating voltage (5–10 kV) is recommended for identifying the subcellular ultrastructure at higher magnification, and a higher accelerating voltage (15–20 kV) is recommended for observing the cell/tissue architecture at lower magnification.

## Advancement of Low-vac SEM in biomedical science and clinical diagnosis

Paraffin and cryostat sections remain widely used in biomedical science and clinical diagnosis for light-microscopic exams because they are affordable and simple to prepare for sectioning. Recent progress in ultrastructural analysis has involved using low-vacuum conditions to reduce negative charge accumulation on non-conductive paraffin or cryostat tissue sections. Low-vac SEM analysis has made significant advancements in the ultrastructural assessment of renal biopsy specimens. Assessing morphological changes in podocytes is crucial for elucidating disease pathogenesis and achieving accurate diagnoses.

Low-vac SEM visualized the spike formation on the glomerular basement membrane (GBM) and alteration of podocyte processes in the membranous nephlopathy combined with platinum blue (Pt-blue) or periodic acid silver-methenamine (PAM) staining [9–11]. In paediatric nephrotic syndrome (NS), ultrastructural differences were recognized between steroid-sensitive and steroid-resistant NS [12]. Briefly, many round-shaped podocytes and elongated primary foot processes were exclusively recognized in steroid-resistant NS, although irregularities in foot process interdigitation, fusions, effacements and microvillus transformations were observed in both steroid-sensitive and steroid-resistant paediatric NS. In IgA nephropathy, the GBM exhibited an etched image and multiple holes in a widening and wavy GBM under Low-vac SEM [13]. Alport syndrome and thin basement membrane nephropathy are genetic disorders caused by mutations of the type IV collagen genes. In this context, Low-vac SEM identified characteristic coarse meshwork appearances in Alport syndrome, and thin and sheet-like appearances of the GBM in thin basement membrane nephropathy [14,15]. In nail-patella syndrome, a rare autosomal dominant disorder, Low-vac SEM revealed coarse meshwork structure and a ragged pattern of the GBM similar to the alterations in Alport syndrome [16].

Transplant glomerulopathy is strongly associated with antibody-mediated rejection and reduced graft survival. Low-vac SEM identified characteristic coarse meshwork structure and duplication of the GBM at the electron microscopic level [17]. In a case of nephrocalcinosis, Low-vac SEM equipped with energy-dispersive X-ray spectrometry verified widespread spherical crystalline deposits primarily composed of calcium phosphate [18]. Methodologically, combined use of Alcian blue and PAM staining was introduced to visualize the endothelial glycocalyx in renal biopsy specimens associated with endothelial injury [19].

Besides the nephropathy, Imazato *et al.* [20] applied Low-vac SEM to cryo-fixed specimens of rat deep fascia and proposed a stratified composition at ultrastructural level. In ophthalmology, Low-vac SEM has been applied to experimental models of injured eyes [21–25]. Arima *et al.* [23] reported progression of corneal wound healing and neovascularization depicted by Low-vac SEM in a rat corneal alkali-burn model. Suppression of macrophage pseudopodia formation was also identified by Low-vac SEM in the experimental model [24]. Takahashi *et al.* [25] revealed ultrastructural disruption of Zinn’s zonule accompanied by inflammatory macrophage-migration in alkali-injured anterior eyes. In a case report of cardiovascular disease [26], three-dimensional ultrastructure of calcified nodules was clearly shown in histopathological paraffin sections by Low-vac SEM, demonstrating distinctive networks of fibrin fibres and platelets capturing erythrocytes in the calcified nodules formed in the thrombus.

## KMnO_4_/Pb metal staining allows uranium free Low-vac SEM imaging

In electron microscopy, SEM produces high-resolution images by scanning a specimen’s surface with a focused electron beam. The resulting reflected or backscattered electrons are generated through elastic scattering interactions with the specimen atoms. Thus far, combined staining with uranyl acetate (Ur) [27] followed by lead citrate (Pb) [28] has been widely used for electron microscopy because heavy elements enhance electron backscatter. However, legal restrictions on radioactive uranyl compounds limit the broader use of three-dimensional imaging via Low-vac SEM. To address this problem, several reports have introduced compounds as substitutes for Ur/Pb metal staining, such as Pt-blue [29], oolong tea extract [30], samarium triacetate [31], gadolinium triacetate [31] or lanthanide salts [32]. but these compounds are originally introduced for transmission electron microscopy of ultrathin sections and not satisfactory for Low-vac SEM.

**Fig. 3.**
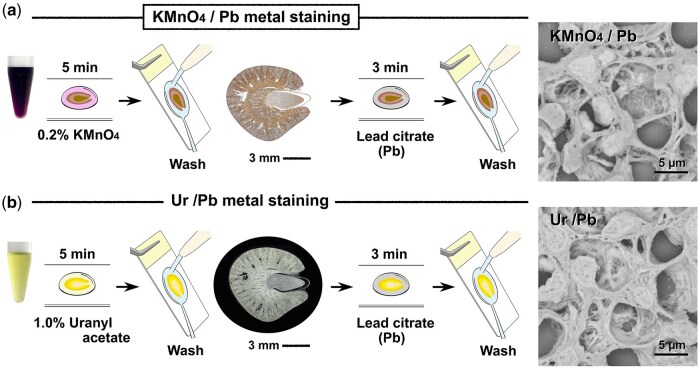
Illustration of KMnO_4_/Pb metal staining and conventional Ur/Pb metal staining. (a) Oxidative treatment with 0.2% KMnO_4_ followed by the application of Reynold’s lead citrate solution. (b) Conventional metal staining with 1.0% uranyl acetate followed by Reynold’s lead citrate solution. Specimens were fixed with a mixture of 2% paraformaldehyde (PFA) and 2.5% glutaraldehyde (GTA) in 0.1 M phosphate buffer (pH 7.4: PB) and prepared as described in the previous publication [37].

**Fig. 4.**
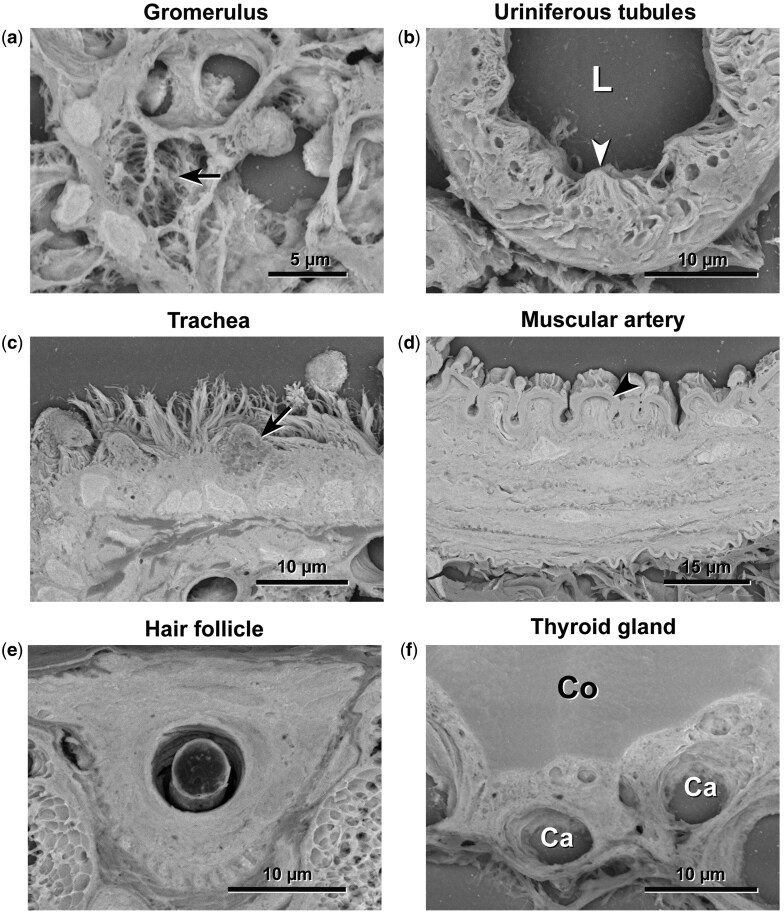
Representative Low-vac SEM micrographs of rat organs by KMnO_4_/Pb metal staining. (a) Glomerulus in the renal corpuscle. Note the fine processes of podocytes (arrow). (b) Cuboidal epithelium of the uriniferous tubules lined with numerous microvilli (arrowhead). L: lumen. (c) Ciliated epithelium in the trachea. Arrow: secretory cells filled with granules. (d) Muscular artery in the kidney. Arrowhead: internal elastic lamina. (e) Hair follicle in the skin. (f) Follicular epithelium and colloid (Co) in the thyroid gland. Ca: capillary lumen. Specimens were fixed with a mixture of 2% PFA and 2.5% GTA in PB and prepared as described in the previous publication [37].

Historically, potassium permanganate (KMnO_4_) oxidation was first introduced in 1956 for fixation to increase electron density [33] and was subsequently utilized for heavy metal staining to enhance image contrast in electron microscopy [34,35]. Next, KMnO_4_ oxidation was previously used to pre-treatment of Lowicryl K4M ultrathin sections for contrast enhancement in immunocytochemistry [36] and was recently utilized for heavy metal staining to enhance image contrast in Low-vac SEM (Fig. 3) [37]. Briefly, the sections were treated with 0.2% KMnO_4_ for 5 min followed by Reynold’s lead citrate solution for 3 min (KMnO_4_/Pb metal staining). The obtained image contrast was equivalent to that of Ur/Pb staining and sufficient for ultrastructural observation, showing the fine processes of podocytes in the glomerulus (Fig. 4a), the uriniferous tubules lined with numerous microvilli (Fig. 4b), the ciliated epithelium in the trachea (Fig. 4c), the internal elastic lamina in muscular artery (Fig. 4d), the hair follicle in the skin (Fig. 4e) and the follicular epithelium in the thyroid gland (Fig. 4f) which were invisible by light microscopy.

## Multiscale Low-vac SEM imaging and CLEM analysis by KMnO_4_/Pb metal staining

**Fig. 5.**
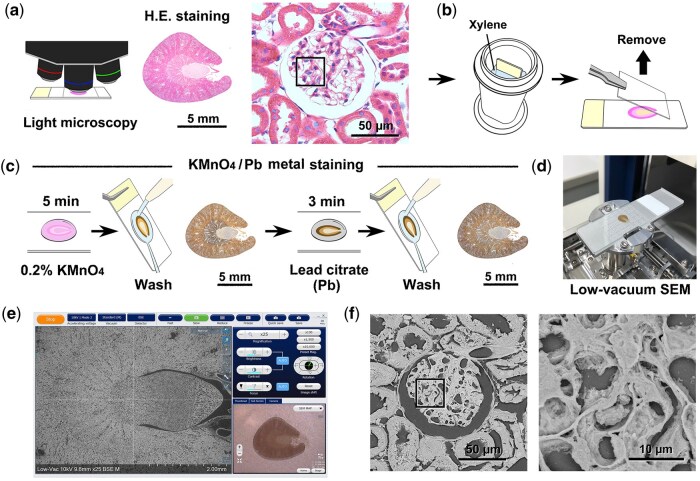
Practical flow diagram of correlative light microscopy and Low-vac SEM imaging by KMnO_4_/Pb metal staining. (a–f) Procedure for correlative light and electron microscopy. (a) Light microscopic survey by haematoxylin and eosin staining. (b) Removal of the coverslip. (c) Oxidative treatment with 0.2% KMnO_4_ followed by Reynold’s lead citrate solution. (d) Setting onto the slide-glass holder. (e) The operation screen combined with the camera navigation window (lower right). (f) Low-vac SEM images of the glomerulus (compare to the light micrograph in (a)). Specimens were fixed with a mixture of 2% PFA and 2.5% GTA in PB and prepared as described in the previous publication [37].

**Fig. 6.**
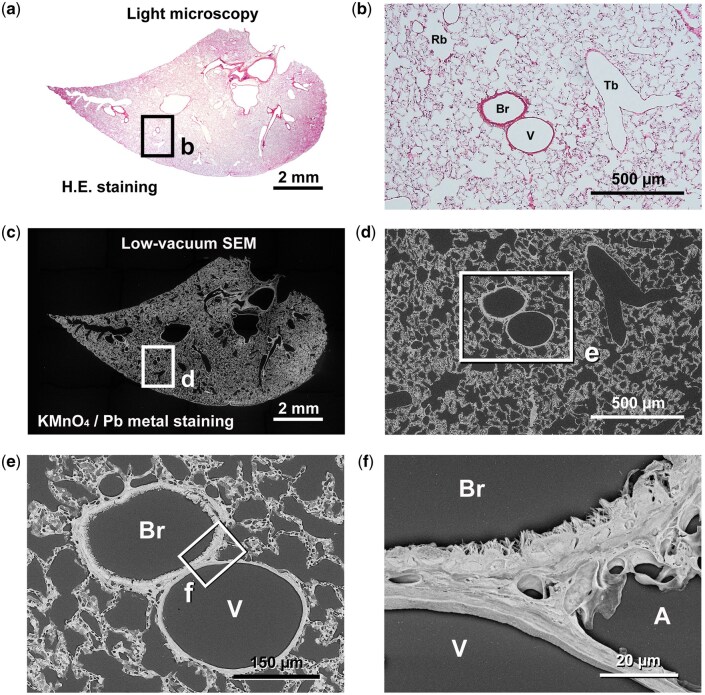
Multiscale correlative light microscopy and Low-vac SEM images of rat lung after KMnO_4_/Pb metal staining. Overview of rat lung by light microscopy (a, b) and Low-vac SEM imaging after KMnO_4_/Pb metal staining corresponding to the light micrographs (b, d). (e, f) Distinct triad ultrastructure of the ciliated cuboidal epithelium of the bronchiole (Br), simple squamous endothelium of the blood vessel (V), and the alveoli with thin capillary vessels (A). Tb, terminal bronchiole; Rb, respiratory bronchiole. Specimens were fixed with a mixture of 2% PFA and 2.5% GTA in PB and prepared as described in the previous publication [37].

Bridging light and electron microscopy, correlative light and electron microscopy (CLEM) [38] has been established to determine the orientation of complex cell/tissue architectures by light microscopy and identify the ultrastructural correlations on the identical paraffin section via Low-vac SEM [11,13,16,17,19,25]. Figure 5 illustrates the flow diagram of multiscale Low-vac SEM, from the centimetre-scale light microscopic survey to the nanometre-scale correlative Low-vac SEM imaging of the rat renal corpuscle by KMnO_4_/Pb metal staining (Fig. 5a and f). After the light microscopic survey by haematoxylin and eosin staining (Fig. 5a), the microscope slides were incubated in xylene for 18–24 h at room temperature to remove the coverslips (Fig. 5b). Then, the sections were treated with 0.2% KMnO_4_ for 5 min followed by Reynold’s lead citrate solution for 3 min (Fig. 5c) and subjected to Low-vac SEM (Fig. 5d). The operation screen combined with the camera navigation window assists in seamless light and electron microscopy observation (Fig. 5e). The KMnO_4_/Pb metal staining technique enables CLEM analysis, making it possible to observe a triad—composed of the ciliated cuboidal epithelium of the bronchiole, endothelium of blood vessels and alveolar capillaries—ranging from light microscopy (Fig. 6a and b) to nano-scale electron microscopy (Fig. 6c–f). Multiscale imaging, from centimetres to nanometres, enhances the scientific reliability of electron micrographs compared to light micrographs.

**Fig. 7.**
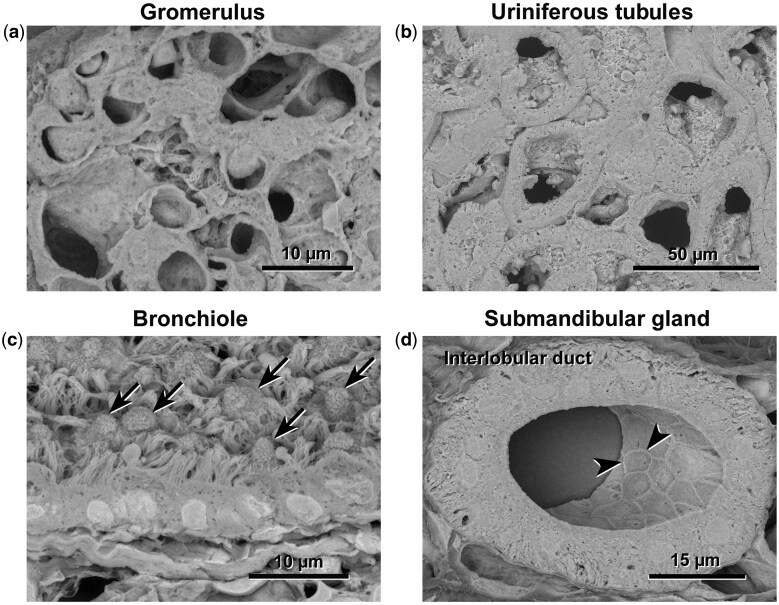
Three-dimensional cell/tissue architectures in 20-µm-thick sectioned organs. Representative Low-vac SEM micrographs of 20-µm-thick sections after KMnO_4_/Pb metal staining. (a) Glomerulus in the renal corpuscle. (b) Cuboidal epithelium of the uriniferous tubules lined with the microvilli (arrows). (c) Ciliated epithelium of bronchioles in the lung. Note the exocrine cells among the ciliated cells. (d) Interlobular duct in the submandibular gland exhibiting the cell boundary (arrowhead) in the luminal face. Specimens were fixed with a mixture of 2% PFA and 2.5% GTA in PB and prepared as described in the previous publication [37, 40].

**Fig. 8.**
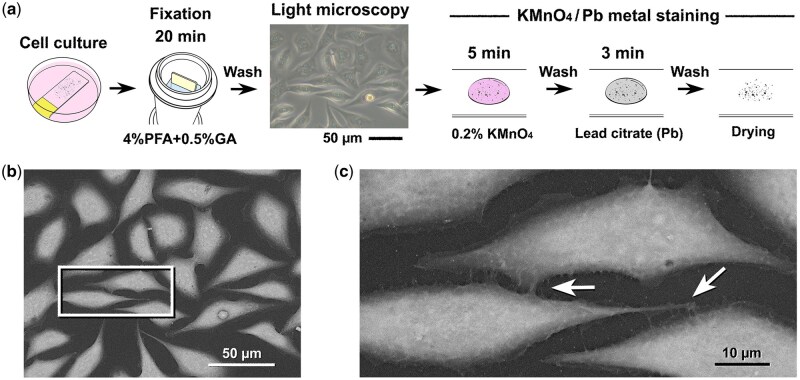
Multiscale correlative light microscopy and Low-vac SEM images of a human pancreatic cancer cell line, SUIT-2, cultured on microscope slides. (a) Illustration of the basic workflow. After cultivation, the SUIT-2 cells were fixed with a mixture of 4% PFA and 0.5% GTA in PB for 30 min. After washing with distilled water, the SUIT-2 cells were subjected to phase contrast light microscopy followed by KMnO_4_/Pb metal staining for Low-vac SEM imaging. (b) Correlative LvSEM image after KMnO_4_/Pb metal staining (compared to the light micrograph in a). (c) High-power view. Note the cell-to-cell connections via the lamellipodia and filopodia (arrows).

The images obtained from 5-µm-thin sections are essentially two-dimensional, making it challenging to reconstruct three-dimensional cell/tissue architectures by examining multiple sections [39]. Further application of Low-vac SEM to thick paraffin/cryostat sections (>15 µm in thickness) accelerates these biomedical challenges by revealing the three-dimensional cell/tissue architectures (Fig. 7) [37,40]. Observation of the 20-µm-thick section facilitates distinctive perception of the face-side (instead of sectioned) images of the epithelium, which are seldom seen within thin sections (Fig. 7b and d). Figure 8 displays KMnO_4_/Pb metal staining of a human pancreatic cancer cell line, SUIT-2 [41], cultured on microscope slides. Correlative light and Low-vac SEM imaging enabled pinpoint targeting observations (Fig. 8a and b). The high-power view revealed cell-to-cell connections via the lamellipodia and filopodia (Fig. 8c).

## *In situ* strategy for biomedical target localization via nanogold nucleation and secondary growth

Visualization of the exact location of targeting molecules presents unique spatial information is called immunocytochemistry, a combination of immunochemistry and cell morphology, which uses specific antibodies against the target molecules of interest. Modern biologists employ immunocytochemistry and advanced fluorescence microscopy, like two-photon excitation, but limited spatial resolution means electron microscopy is often required to answer key questions [1,2].

Gold nanoparticles with diameters ranging from 1 to 100 nm are referred to as colloidal gold when suspended in aqueous solutions. Faraday first described the optical properties of nanoscale gold in 1857, using reduced chloroauric acid [42]. Following the Turkevich’s pioneering study [43] in 1951, various experimental methods have been developed for the synthesis of nanogold particles which identified the nucleation and growth process in the synthesis of colloidal gold. In 1959, Singer [44] introduced the use of antibodies conjugated with the electron-dense protein ferritin for immunocytochemistry. This advancement addressed the limitation of fluorescent probes, whose electron-scattering properties are inadequate for effective electron microscopy. Following the publication by Faulk and Taylor in 1971 [45], the standard approach to electron microscopic localization has involved conjugating nanogold probes to antibodies [46,47].

Nanogold probes can be easily identified and counted on labelled cells or tissues for quantitative analysis of labelling intensity. However, standard methods involve lengthy preparation of nanogold probes [48,49] and antibody conjugation [46,47]. In post-embedding nanogold probe labelling, larger particles reduce labelling intensity [50], yet particles over 30 nm are essential for Low-vac SEM imaging. To resolve this problem, an innovative technique has been established that incorporates ‘*in situ* nanogold nucleation’ within diaminobenzidine (DAB) products deposited at the target molecules on universal paraffin/cryostat sections [51]. The proposed procedure employs the catalytic properties of an enzyme (horseradish peroxidase) that yields a red/brown-coloured reaction product from DAB [52]. Unlike immunofluorescence, the enzyme system [53] produces permanent precipitates visible with a standard bright-field microscope.

**Fig. 9.**
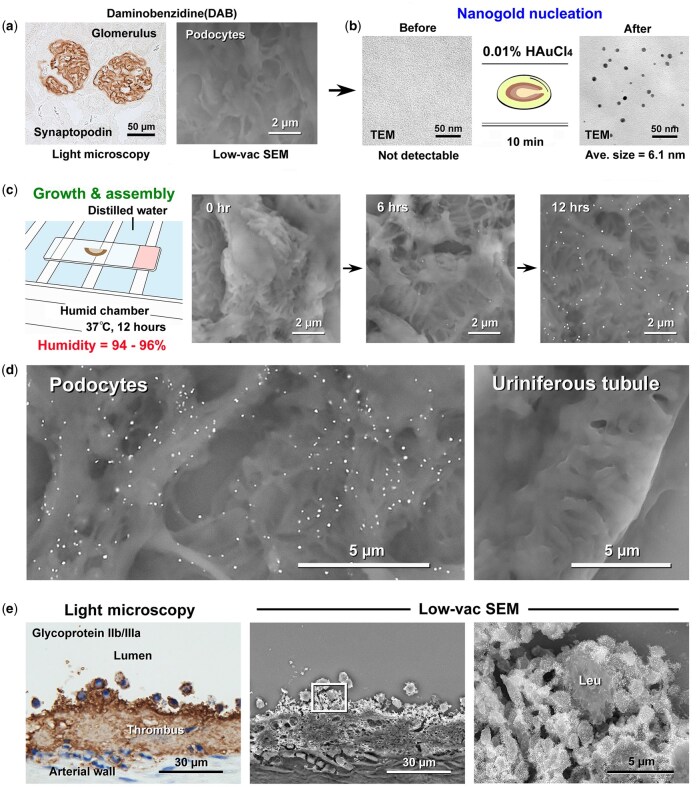
Diagram of *in situ* nanogold labelling for Low-vac SEM target localization. (a) The DAB deposition sites for the target molecules (e.g. synaptopodin) surveyed under a light microscope. (b) Ultrathin transmission electron microscope (TEM) images show nanogold particles nucleated *in situ* among the DAB deposition sites after treatment with 0.01% HAuCl_4_ for 10 min. (c) Time-lapse secondary growth and assembly in hot-humid conditions. Note the satisfactory labelling after 12 h for Low-vac SEM target localization. (d) Specific labelling on the processes of podocytes in the kidney glomerulus (left). The cuboidal epithelium of the uriniferous tube, as a negative control, shows no particles (right). (e) Pathological application to rabbit arterial thrombosis. Note the intense labelling of glycoprotein IIb/IIIa expressed on the platelets surrounding the leukocyte (Leu). The cell nuclei were stained with haematoxylin for light microscopy. Specimens were prepared as described in the previous publication [51].

Figure 9 illustrates the flow diagram *in situ* nanogold labelling for Low-vac SEM target localization (in this experiment, synaptopodin [54], expressed in the process of podocytes in the kidney glomerulus). After the light microscopic survey (Fig. 9a), the sections were treated with 0.01% tetrachloroauric acid (HAuCl_4_) for 10 min, leading to nanogold particle ‘nucleation’ (Fig. 9b). Then, the sections were exposed to hot-humid air in a humid chamber at 37 °C for 9–15 h to accomplish the ‘secondary growth and assembly’ of the nucleated nanogold particles around the target molecules (Fig. 9c) [51].

Nanogold particle formation is explained by LaMer’s classic theory and its updates, which describe burst nucleation as the initial phase transition step [55]. The nucleated particles serve as ‘seeds’ for secondary growth through monomer addition, aggregation and coalescence. As shown in the representative electron micrographs (Fig. 9d), nanogold particles were specifically localized on the process of podocytes in the kidney glomerulus, consistent with the light microscopic findings. The fine structural details of podocytes and their cellular processes, previously undetectable by conventional light microscopy, were distinctly visualised using Low-vac SEM. The pathological application showed intense labelling of platelet glycoprotein IIb/IIIa in rabbit arterial thrombosis (Fig. 9e) [56]. *In situ* nanogold labelling was used in the histopathological study of Creutzfeldt–Jakob disease [57] and the investigation of deep venous thrombus formation [58] examined with Low-vac SEM.

## Future perspective

Advances in biomedical research, such as 3D organoid generation from iPS cells [59–61] and CRISPR/Cas9-induced morphological changes [62,63], require three-dimensional analysis of cell and tissue architecture and ultrastructural examination of gene- or molecule-specific phenotypes. The user-friendly Low-vac SEM techniques, KMnO_4_/Pb metal staining and *in situ* nanogold labelling, discussed here are expected to bridge the gap between light and electron microscopy for correlating cell or tissue structure and function. Paraffin and cryostat blocks of cell or tissue samples are semipermanent [64], which makes them useful for retrospective analysis of archived specimens. Expanding Low-vac SEM imaging could introduce innovative methodologies in the field of biomedical electron microscopy, benefiting clinical research and diagnostics.
